# A novel method to rapidly distinguish the geographical origin of traditional fermented-salted vegetables by mass fingerprinting

**DOI:** 10.1371/journal.pone.0188217

**Published:** 2017-11-17

**Authors:** So-Ra Yoon, Sung Hyun Kim, Hae-Won Lee, Ji-Hyoung Ha

**Affiliations:** Hygienic Safety and Analysis Center, World Institute of Kimchi, Gwangju, Republic of Korea; University of Helsinki, FINLAND

## Abstract

The geographical origin of kimchi is of interest to consumers and producers because the prices of commercial kimchi products can vary significantly according to the geographical origin. Hence, social issues related to the geographical origin of kimchi in Korea have emerged as a major problem. In this study, the geographical origin of kimchi was determined by comparing the mass fingerprints obtained for Korean and Chinese kimchi samples by MALDI-TOF MS with multivariate analysis. The results obtained herein provide an accurate, powerful tool to clearly discriminate kimchi samples based on their geographical origin within a short time and to ensure food authenticity, which is of significance in the kimchi industry. Furthermore, our MALDI-TOF MS method could be applied to determining the geographical origin of other fermented-salted vegetables at a reduced cost in shorter times.

## Introduction

The identification of the geographical origin of food products is an international issue. In particular, the deliberate false labelling and adulteration of food products is known to be a problem of increasing global interest [1]. Therefore, systems that indicate the origin of traditional foods have been widely applied worldwide to ensure excellence by country. For example, the European Union is encouraging the use of the Traditional Speciality Guaranteed (TSG) label to protect and improve traditional food labelling [2, 3].

Kimchi is a traditional fermented-salted Korean side dish made of vegetables, typically prepared by fermenting napa cabbage (*Brassica rapa* subsp. *pekinensis*) with various seasonings, e.g. red pepper, onion, garlic, and ginger. Kimchi is the most widely consumed item in Korea and has been consumed by Koreans for approximately 2,000 years [4]. In 2001, kimchi was listed in the Codex Alimentarius (CODEX STAN 223–2001). Further, kimchi has been reported to be a good source of probiotics, as it contains lactic acid bacteria [5].

In Korea, the geographical origin of kimchi has emerged as a major problem for both consumers and producers because the prices of commercial kimchi can vary according to its geographic origin. Since 2008, the Korean Ministry of Agriculture, Food and Rural Affairs has made it mandatory for the food service sector to state the origin of prepared kimchi samples; however, cases of rule violations have begun to increase [6]. In particular, an increasing number of cases related to the false labelling of Chinese kimchi as Korean kimchi by unscrupulous traders in Korea have been reported. With the increase in such cases, consumers have demanded severe penalties for false designations of origin, with a desire to accurately know the origin of kimchi [6]. Hence, an analytical technique for discriminating between Chinese and Korean kimchi is crucial in Korea. However, such analytical methods have been rarely studied and are difficult to apply to kimchi, as it is necessary to analyse all of the ingredients in kimchi. Previous studies have focused on identifying elements, biochemical compositions, and metabolites of kimchi extracts using nuclear magnetic resonance (NMR) spectroscopy or strontium isotope ratio measurements [7, 8]. In addition, DNA technology, e.g. polymerase chain reaction (PCR), which has a high sensitivity, can rapidly and conveniently identify the geographical origin of foods, such as mozzarella cheese [9], grapes [10], physalis fruits [11], and Shea tree fruits [12]. However, for food samples of the same species from different regions, this methodology cannot discriminate samples based on geographical origin.

Typically, matrix-assisted laser desorption ionization time-of-flight mass spectrometry (MALDI-TOF MS) has been employed for identification via the analysis of unique spectra or fingerprints in biomarker discovery [13], food authorization [1, 14], and microorganism identification [15, 16]. In the food industry, MALDI-TOF MS has been effectively used to determine the geographical origin or species of food, e.g. honey [1], mushrooms [17], shrimp [18], scallops [19], meat, and gelatin [14], owing to its advantages, e.g. accuracy, speed, user-friendliness, and high sensitivity, even for low-molecular-weight samples [20]. However, to the best of our knowledge, mass fingerprinting by MALDI-TOF MS has not been previously investigated to determine the geographical origin of kimchi. Thus, a MALDI-TOF MS approach was used to discriminate between Korean and Chinese kimchi samples based on geographical origin. Based on environmental differences between the two countries, even if an ingredient in kimchi originating from the same species has the same DNA genome sequence, protein expression could differ according to the environment of growth and development.

In this study, the geographical origin of kimchi, which is a complex food matrix mixed with seasoning, was determined by comparison of the mass fingerprints of Chinese and Korean kimchi samples using MALDI-TOF MS. This approach for identifying biomarkers can be used as a rapid, reliable tool to discriminate between kimchi samples of different origins.

## Materials and methods

### Sample preparation

Twenty kimchi samples, made from Korean or Chinese kimchi cabbage, were acquired via online markets. Korean kimchi samples (n = 10) were purchased in May 2016, with labels indicating various regions (Gyeonggi, Chungcheongbuk, Gyeongsangbuk, Gyeongsangnam, Jeollanam, and Gangwon provinces) in South Korea, and Chinese kimchi samples (n = 10) were purchased in the same month, with labels indicating Shandong Province in China. Each head of kimchi cabbage was cut into eight equal piece. The cut kimchi samples were stored in sealed bags and fermented for 1, 2, 3, and 4 weeks at 4°C. After fermentation, the samples were frozen using liquid nitrogen to prevent the loss of proteins, and then ground using an electronic blender. The ground kimchi samples were stored at ‒80°C until MALDI-TOF MS analysis.

### MALDI-TOF MS analysis

MALDI-TOF MS analysis was performed in triplicate for kimchi samples without protein extraction. First, kimchi samples were mixed and homogenized in a 1:1 ratio using α-cyano-4-hydroxycinnamic acid (CHCA) dissolved in 30% acetonitrile (ACN) containing 0.1% trifluoroacetic acid (TFA). The homogenized samples with the sample matrix were dried in air and deposited on a MALDI target plate at room temperature. Mass spectra in the mass range from 2,000 to 20,000 m/z were obtained in the linear and positive-ion modes with a laser frequency of 100 Hz using an UltrafleXtreme TOF/TOF mass spectrometer (Bruker Daltonics, Germany) installed at the Daedeok Headquarters, Korea Basic Science Institute (KBSI). The extraction delay time, lens, and ion sources 1 and 2 were set at 250 ns, 7, 25, and 23.6 kV, respectively. Mass spectral data were acquired in the mzXML format using Flexcontrol and Flexanalysis software (Bruker Daltonics, Germany). Calibration for MALDI-TOF MS analysis was carried out using insulin ([M+H]^+^, 5,734.51 m/z), ubiquitin I ([M+H]^+^, 8,565.76 m/z), cytochrome C ([M+H]^+^, 12,360.97 m/z; [M+H]^2+^, 6,180.99 m/z), and myoglobin ([M+H]^+^, 16,952.30 m/z; [M+H]^2+^, 8,476.65 m/z) from the protein calibration standard I kit (Bruker Daltonics, Germany).

### Mass fingerprinting analysis

Mass fingerprinting analysis of the spectra acquired from kimchi samples was performed using Mass-Up open software [21]. Pre-processing for calibration and alignment between the mass spectra of the samples was performed by intensity transformation (none), smoothing (none), baseline correction (Snip algorithm), and standardization (total ion current). In addition, peak detection was performed with the MALDIquant package [22], with a signal-to-noise ratio of 3 and a half-window size of 60. Peaks were matched with the following parameters: i) Intra-sample matching (MALDIquant: tolerance (0.002), without selecting the generate consensus spectrum box); and ii) Inter-sample matching (MALDIquent: tolerance (0.002)). In addition, MALDIquant, which is a multifaceted statistical package for analysing MALDI-TOF MS spectra, was used to repetitively perform complex analysis for pre-processing, non-linear peak alignment, and calibration of the raw mass spectral data.

Principal component analysis (PCA) of the mass fingerprints was performed using the pre-processed mass spectral data. The maximum component and variance covered values were set to 0 and 0.95, respectively. In addition, hierarchical clustering analysis (HCA) was performed using the pre-processed mass spectral data, and the minimum variance, cluster reference, distance function, conversion, intra-sample minimum presence, and deep clustering values were set to 0, the farthest, Euclidean, percentage of presence, 0, and no, respectively.

The discriminated mass peaks separated as Chinese and Korean kimchi were obtained by the Benjamini Hochberg FDR method [21] considering a *q*-value < 0.2 using the biomarker discovery (inter-class analysis) mode in the Mass-Up software. The heat map based on the discriminated mass peaks was prepared using MultiExperiment Viewer (MeV) [23].

## Results

### MALDI-TOF MS analysis of kimchi samples

The MALDI-TOF MS spectra of kimchi fermented for 1, 2, 3, and 4 weeks were obtained in the mass range of 2,000–20,000 m/z; in total, 80 spectra were recorded in triplicate. However, the mass range of the actual acquired mass spectra of kimchi samples was set to 2,000–10,000 m/z. Mass peaks greater than 10,000 m/z were not detected in all kimchi samples. PCA and HCA of these spectra were carried out using the Mass-Up software.

### Discrimination of kimchi by fermentation time based on geographical origin using PCA

**Fig 1:**
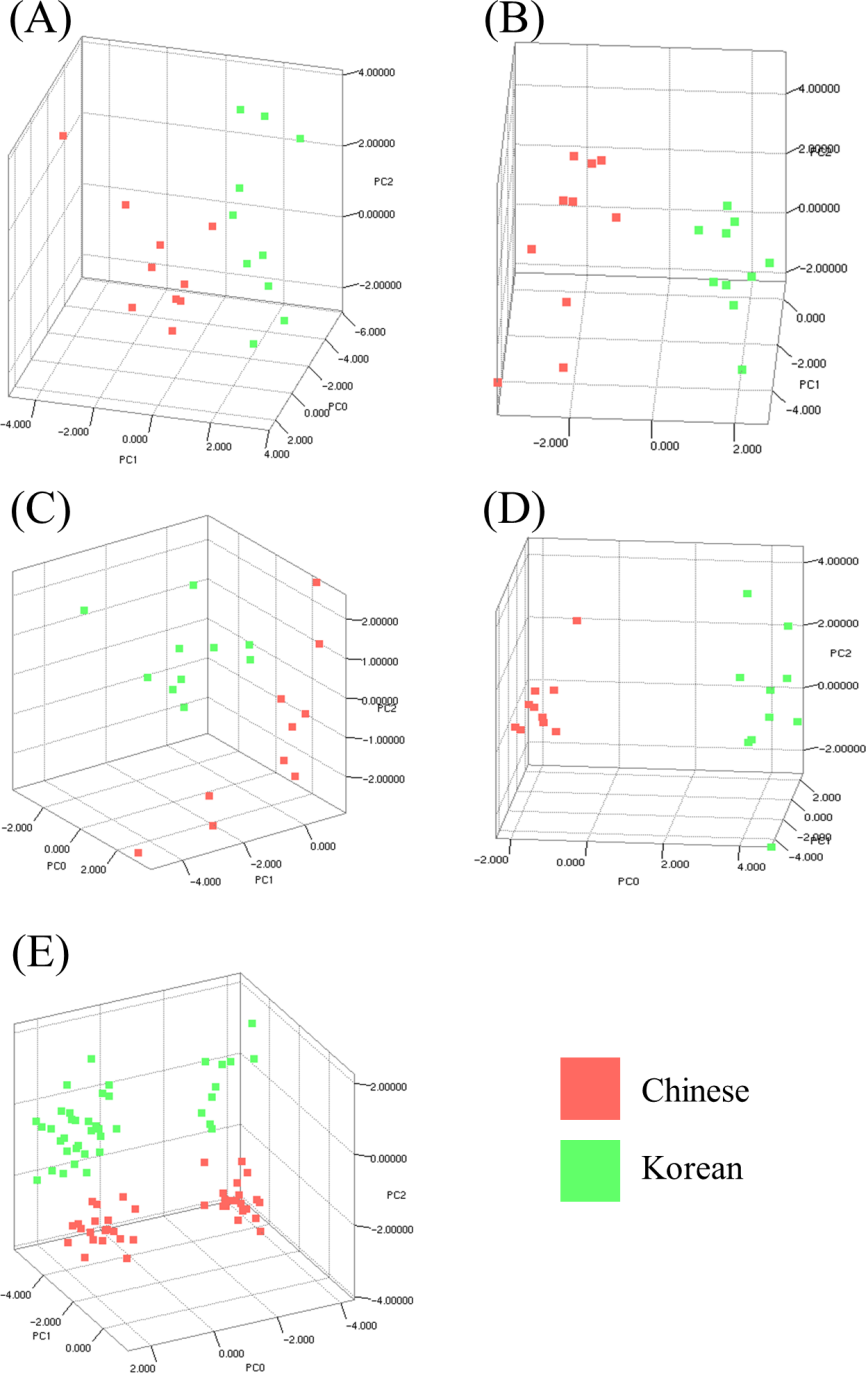
PCA of the mass spectra of Korean and Chinese kimchi during fermentation using the Mass-Up software. The viewpoints of the PCA scatter plots in this figure were selected to show discrimination between the Chinese and Korean kimchi samples. PCA was performed based on the mass spectra acquired for Chinese and Korean kimchi after fermentation for 1 (A), 2 (B), 3 (C), and 4 (D) weeks, and throughout fermentation (E). Red squares, Chinese kimchi; green squares, Korean kimchi.

To visualize differences and similarities between Chinese and Korean kimchi, PCA, a multivariate analysis approach, was conducted using each mass spectrum acquired from 20 kimchi samples (Korean: n = 10 and Chinese: n = 10) according to fermentation time (1, 2, 3, and 4 weeks, and throughout fermentation; Fig 1 and S1 Fig). To examine correlations, the principal component (PC) scores were expressed in three-dimensional scatter plots (PC0, PC1, and PC2 plotted on x, y, and z axes, respectively). Fig 1(A) shows the sample score plots for PC0 vs PC1 vs PC2 after fermentation of the Chinese and Korean kimchi samples for 1 week. The top and right sides of the 3D scatter plot indicated that three Chinese kimchi samples belong to the Korean kimchi group based on the zero point of PC1, whereas the left side did not indicate any significant observations with respect to PC0, PC1, or PC2 (S1 Fig). On the top side of the sample score plot after fermentation for 2 weeks, the Chinese kimchi samples fermented were more distinguished from the Korean kimchi samples. The left side was divided into two sectors based on the zero point of PC0, similar to the trend observed in the top side, whereas the right side was not divided (Fig 1(B) and S1 Fig). As shown in Fig 1(C), on the left side of the scatter plot after fermentation for 3 weeks, the 10 Chinese kimchi samples were located to the left of the PC0 zero point, as well as two Korean kimchi samples, whereas the remaining eight Korean kimchi samples were located to the right of the zero point of PC0 (S1 Fig). On the top and right side of the scatter plot after fermentation for 3 weeks, Chinese kimchi D and Korean kimchi H were positioned relatively far away from the other samples, which are situated to the right of the ‒2 value of PC1, indicating that their mass spectra or part of their mass spectral patterns are different from those of the other samples. That is, all the kimchi samples, except Chinese kimchi D and Korean kimchi H, exhibited partially similar mass spectral patterns. With respect to fermentation time, the distinction between Chinese and Korean kimchi samples was most obvious after fermentation for 4 weeks, except on the right side of the scatter plot, where it was difficult to classify this difference (Fig 1(D) and S1 Fig). Throughout fermentation, the Chinese and Korean kimchi samples were diagonally divided from the upper right to lower left based on the ‒2 value of PC1 (Fig 1(E)).

### Discrimination of kimchi by fermentation phase based on geographical origin using HCA

**Fig 2:**
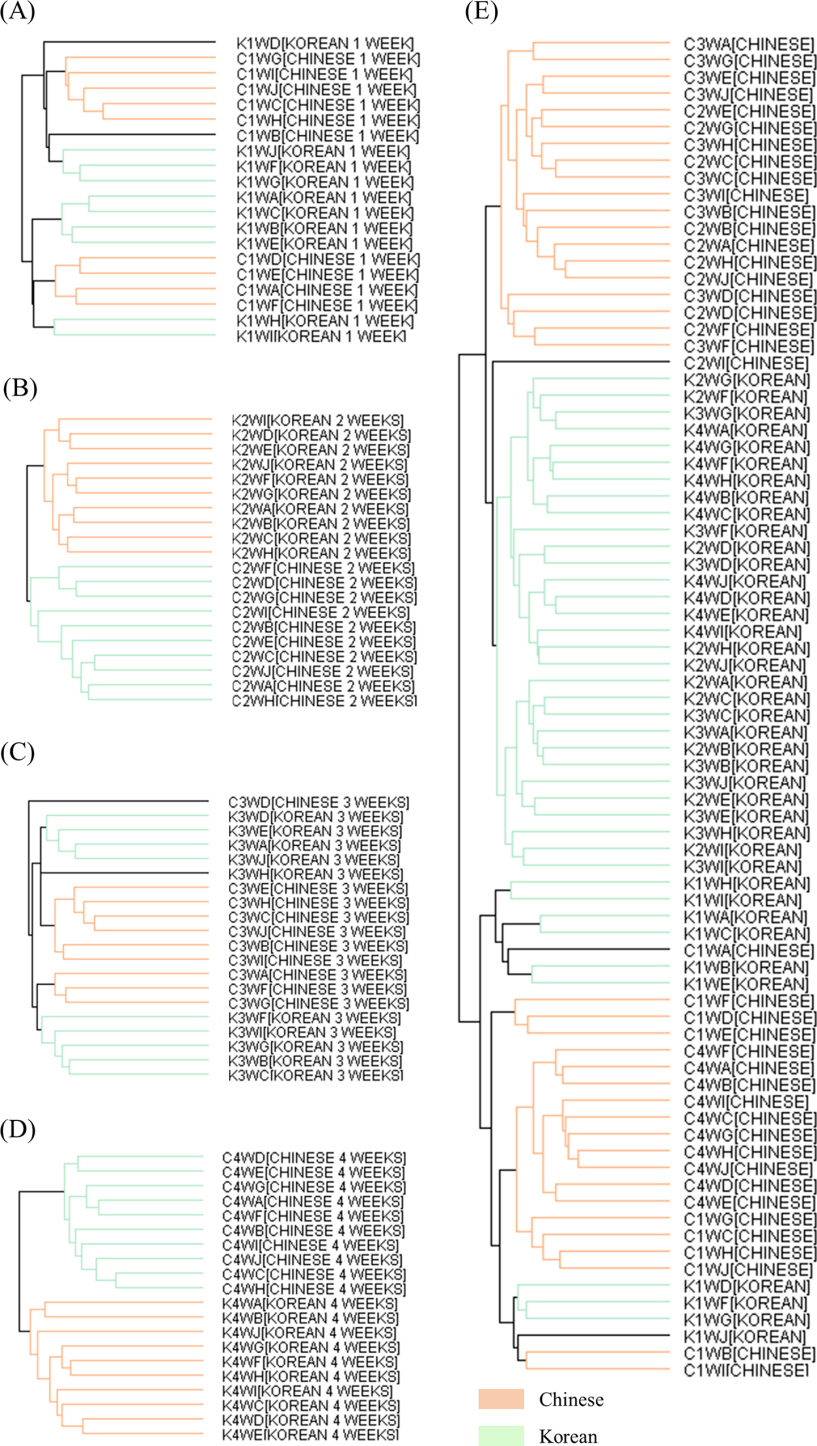
Dendrograms obtained by HCA of the mass spectra of Korean and Chinese kimchi during fermentation using the Mass-Up software. Dendrograms for Chinese and Korean kimchi after fermentation for 1 (A), 2 (B), 3 (C), and 4 (D) weeks, and throughout fermentation (E). The first capital letter of the sample name refers to the country of origin (C, Chinese; K, Korean). The second number and third capital letter of the sample name refer to the fermentation time (1W to 4W, fermentation for 1 week to 4 weeks). The fourth capital letter of the sample name refers to the kind of kimchi. Orange lines, Chinese kimchi; green lines, Korean kimchi.

HCA, which is a multivariate analysis approach different from PCA, was performed to visualize the MALDI-TOF MS mass spectra acquired for 20 kimchi samples (Korean: n = 10 and Chinese: n = 10) according to fermentation time (1, 2, 3, and 4 weeks) (Fig 2). The results of the agglomerative HCA were expressed as dendrograms. After fermentation for 1 week, the Chinese kimchi samples A, D, E, and F belonged to the Korean kimchi group, i.e. these samples were located between Korean kimchi E and H (Fig 2(A)). Thus, Chinese kimchi B and Korean kimchi D were not grouped with the Chinese and Korean kimchi samples, respectively, indicating that the mass spectrum acquired for Chinese kimchi sample B is associated with Korean kimchi samples F, G, and J. In contrast, Korean kimchi sample D is not clearly associated with any of the Korean or Chinese kimchi samples. After fermentation for 2 weeks, the kimchi samples were clearly separated into two clusters, i.e. Korean and Chinese groups (Fig 2(B)). After fermentation for 3 weeks, two individual samples (Korean kimchi H and Chinese kimchi D) and four closely grouped sample clusters were observed. The first sample cluster closely grouped Korean kimchi samples B, C, F, G, and I; the second sample cluster closely grouped Chinese kimchi samples A, F, and G; the third sample cluster closely grouped Chinese kimchi samples B, C, E, H, I, and J; and the fourth sample cluster closely grouped Korean kimchi samples A, D, E, and J (Fig 2(C)). Notably, the sample clusters of Korean kimchi (first and fourth) and of Chinese kimchi (second and third) were not grouped together. Korean kimchi sample H was located between the third and fourth clusters, whereas Chinese kimchi sample D only exhibited marginal correlation with the four clusters. After 4 weeks of fermentation, all the Chinese kimchi samples were clustered in a Chinese only group, whereas all the Korean kimchi samples were clustered in a Korean only group (Fig 2(D)), similar to the dendrogram obtained for the kimchi samples after 2 weeks of fermentation (Fig 2(B)). However, the distance between each sample subjected to fermentation for 2 weeks was not the same as that between each sample subjected to fermentation for 4 weeks. However, in the HCA of the kimchi samples at all fermentation times, the Chinese and Korean kimchi samples were not clustered into separate Chinese and Korean groups (Fig 2(E)).

### Discrimination of kimchi by selected mass peaks based on geographical origin

**Fig 3:**
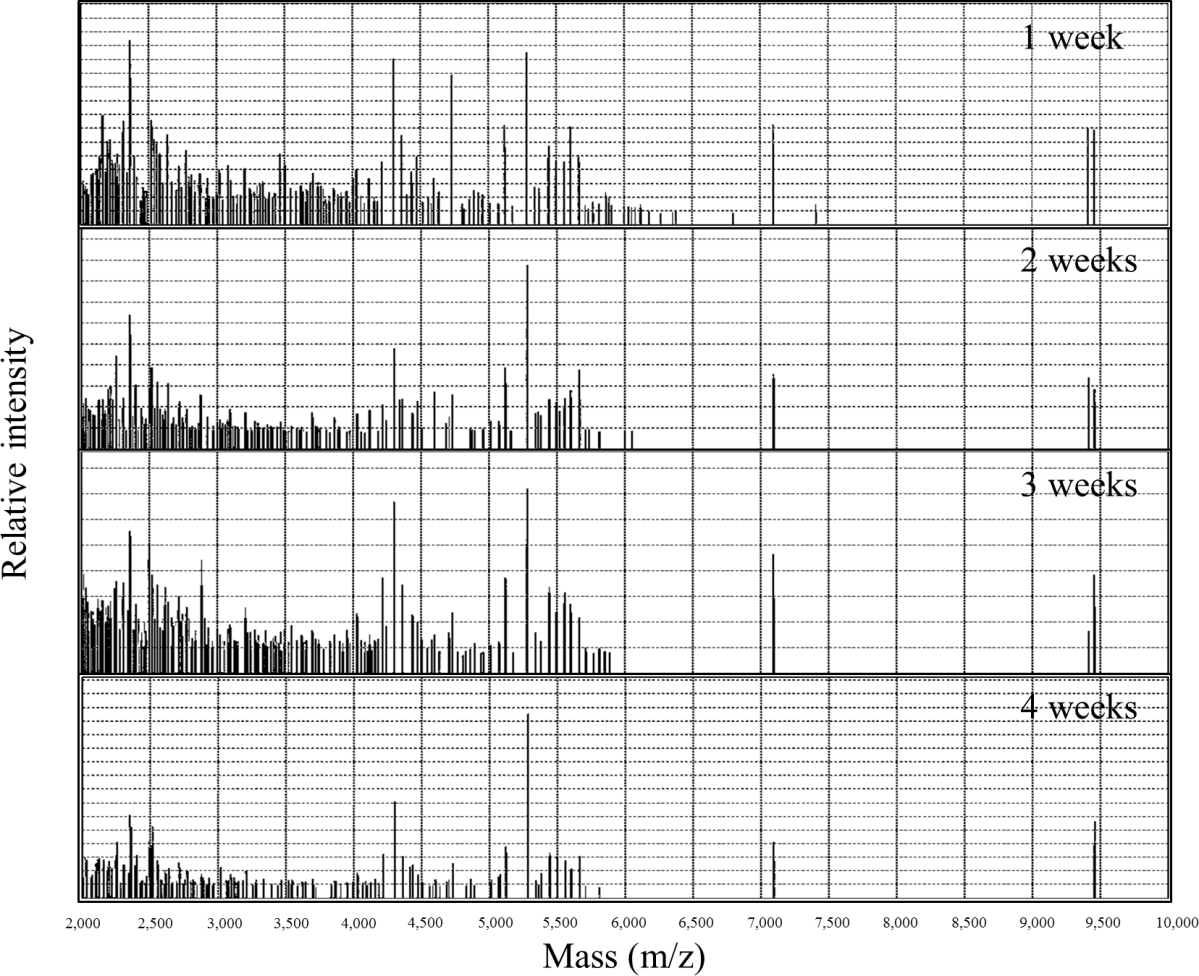
Change in the mass spectra (2,000–10,000 m/z) of Korean kimchi a during fermentation.

**Fig 4:**
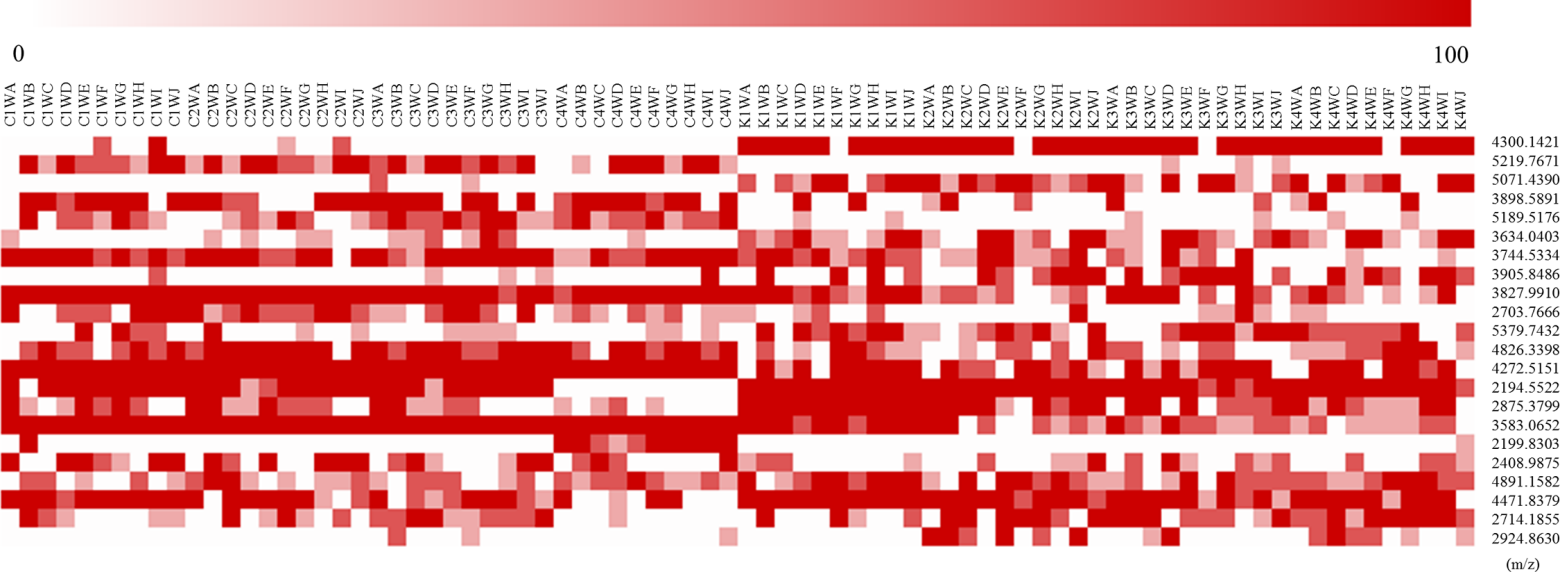
Heat map showing the mass spectra of Chinese and Korean kimchi samples in the mass range of 2,000 to 10,000 m/z (*q*-value < 0.2). The colour intensity of each panel is proportional to the relative intensity of the mass value (max. 100%). The first capital letter of the sample name refers to the country of origin (C, Chinese; K, Korean). The second number and third capital letter of the sample name refer to the fermentation time (1W to 4W, fermentation for 1 week to 4 weeks). The fourth capital letter of the sample name refers to the kind of kimchi.

The MALDI-TOF MS mass spectra of Korean kimchi A indicated that the relative intensity of the discernment peak, which demonstrated potential to distinguish between Korean and Chinese kimchi, increased as fermentation progressed (Fig 3). The mass spectra of the other kimchi samples also indicated the same tendency (S2 Fig). Hence, re-analysis was conducted with significantly different mass peaks (total 22 mass peaks) extracted from the mass spectra (mass range of 2,000–10,000 m/z) of the kimchi samples throughout the fermentation time based on the *q*-value (<0.2). First, to analyse the intensity of each peak extracted from the mass spectra of the kimchi samples, a heat map, expressing the intensity of the mass peaks as colours, was utilized (Fig 4). In this map, higher intensities are associated with darker red colours. Three mass peaks (3827.9910, 4272.5151, and 3583.0652 m/z) were detected in all the Chinese kimchi samples, and one mass peak (2194.5522 m/z) was detected in all the Korean kimchi samples. Interestingly, various other peaks were mainly detected in Chinese or Korean samples, indicating a difference between Chinese and Korean kimchi. For example, after 1 week of fermentation, a peak at 3634.0403 m/z was observed in nine Korean kimchi samples, but in only one Chinese kimchi sample. In addition, after 2 weeks of fermentation, a peak at 2703.7666 m/z was detected in all the Chinese kimchi samples, but in only one Korean kimchi sample. After fermentation for 4 weeks, a distinct at 2194.5522 m/z was only detected in Korean kimchi. In contrast, nine Chinese kimchi samples had a peak at 3898.5891 m/z, but this peak was not observed in nine Korean kimchi samples. Throughout fermentation, peaks at 4300.1421 and 5071.4390 m/z were mostly observed in Korean kimchi samples. In detail, the peak at 4300.1421 m/z was observed in all Korean kimchi samples, except for Korean kimchi F, but was only detected in four Chinese kimchi samples. Further, the peak at 5219.7671 m/z was mainly detected in Chinese kimchi samples rather than in Korean kimchi samples.

**Fig 5:**
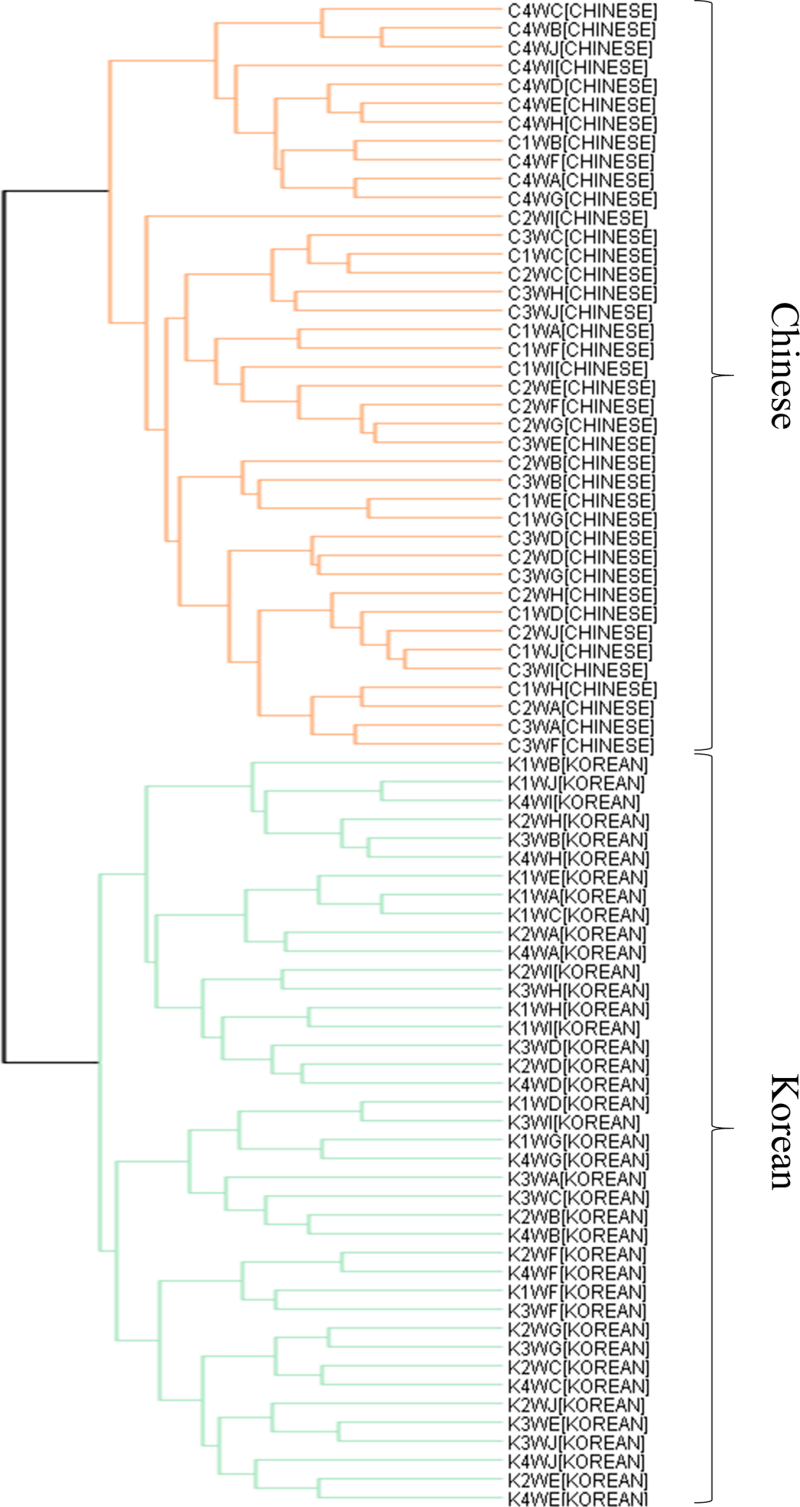
HCA of mass spectral peaks selected by the *q*-value (<0.2) for Chinese and Korean kimchi samples. The first capital letter of the sample name refers to the country of origin (C, Chinese; K, Korean). The second number and third capital letter of the sample name refer to the fermentation time (1W to 4W, fermentation for 1 week to 4 weeks). The fourth capital letter of the sample name refers to the kind of kimchi.

To complement and more clearly visualize the heat map, HCA was conducted based on mass peak discrimination. Two agglomerative hierarchical clusters were observed for the kimchi samples throughout fermentation (Fig 5). All the Chinese kimchi samples were clustered in the Chinese only group, and all the Korean kimchi samples were clustered in the Korean only group.

## Discussion

In this study, MALDI-TOF MS was utilized to discriminate between Chinese and Korean kimchi of different geological origins after subjecting these samples to fermentation for 1, 2, 3, and 4 weeks. To the best of our knowledge, a mass fingerprinting approach using MALDI-TOF MS has not been performed to analyse kimchi samples based on fermentation time. Because kimchi is a fermented food, to discriminate between Chinese and Korean kimchi based on geographical origin, samples must be prepared at various fermentation times to find a reliable biomarker that remains constant during fermentation. To enable analysis within 10 min, a simple kimchi sample preparation method, involving only cutting, freezing with liquid nitrogen, and grinding, was carried out before MALDI-TOF MS analysis. The mass spectra of the kimchi samples obtained by MALDI-TOF MS were analysed using PCA, HCA, and heat maps. PCA, which is a multivariable analysis method, has been applied for the discrimination of coffee [24], cocoa [25], wine [26], saffron [27], and honey [1] based on geographical origin.

As shown in Fig 1, 3D PCA scatter plots showed the variance of Chinese and Korean kimchi samples subjected to fermentation. However, PCA did not reveal clear relationships between kimchi samples or groups. In contrast, the dendrograms obtained by HCA intuitively depicted the relationships among the kimchi samples in terms of distance (Fig 2). In HCA, the kimchi samples that were not separated after 1 week of fermentation were divided into two clusters (Korean and Chinese groups) after 2 weeks of fermentation (Fig 2(B)); however, these clusters were not appropriately separated after 3 weeks of fermentation (Fig 2(C)). In particular, after 1 week of fermentation, Chinese kimchi D and Korean kimchi H were grouped with the Chinese and Korean samples, respectively, but these samples were not grouped after 3 weeks of fermentation. The HCA result obtained after fermentation for 3 weeks was in agreement with that obtained by PCA: Chinese kimchi D and Korean kimchi H were distributed far away from the other samples in the 3D scatter plot in the right and left views (S1 Fig). This result implied that various polymers, such as proteins or polysaccharides, disappeared or appeared, with the amount affected by the fermentation process. The principal component scatter plot of the kimchi samples after 4 weeks of fermentation (Fig 1(D)) showed the best discrimination between the Chinese group and the Korean group as compared with the scatter plots for 1, 2, and 3 weeks of fermentation (Fig 1(A)–1(C)). In addition, in HCA, after fermentation for 4 weeks, all the kimchi samples were clearly clustered into Chinese and Korean groups (Fig 2(D)). These results indicated that their mass spectra or at least part of their mass spectral patterns become similar during fermentation depending on geographical origin. Plants grown in the same environment are likely to exhibit certain uniform patterns throughout fermentation. In addition, the climate and soil components depend on the environment and affect the mass spectra; therefore, plants grown under the same conditions have similar nutrients and metabolites, and it is possible that plants grown in the same environment will exhibit similar mass spectral patterns. In HCA, after fermentation for 4 weeks, the kimchi samples were clearly separated as Chinese and Korean kimchi. This hierarchical clustering was in agreement with the PCA classification after fermentation for 4 weeks, indicating that a particular pattern or component identified by MS spectra may be used to distinguish between Chinese and Korean kimchi after 4 weeks of fermentation. However, when fermentation time was not taken into account, the samples were not separated into Chinese and Korean groups (Fig 2).

Interestingly, as fermentation progressed, HCA was found to discriminate successfully between kimchi samples based on geographical origin (Fig 2), and the mass spectra of samples subjected to fermentation showed a relative increase in the intensity of discernment peaks and a relative decrease in the intensity of other peaks (Fig 3 and S2 Fig). Based on these results, using peaks selected by the *q*-value (<0.2), all the kimchi samples clearly successfully clustered into Chinese and Korean groups, regardless of the fermentation time (Fig 5). The heat map indicates the potential to discriminate between Chinese and Korean samples using specific polymers (Fig 4). This phenomenon is based on the fact that polymers (2,000–10,000 m/z) in kimchi degrade with the progress of fermentation, but the essential polymers for discrimination based on geographical origin are not degraded. Hence, based on this supposition, 22 significantly different peaks in the mass range of 2,000–10,000 m/z with q-values of less than 0.2 were extracted from the mass spectra of the kimchi samples. The degradation of polymers with the progress of fermentation has been reported for sourdough [28] and fermented unsalted soybean paste [29]. Hence, further studies related to discriminating geographical origin by targeting proteomes in fermented foods should consider the degradation of polymers during fermentation. The approach utilized herein can be utilized to identify the geographical origin of kimchi. This approach comprises sample homogenization, MALDI-TOF MS analysis, mass spectra normalization, comparison with existing kimchi mass spectra, peak extraction from mass spectra by *q*-value, identification of position on the HCA dendrogram, and identification of geographical origin of samples. Therefore, constructing a database of mass spectra of kimchi samples, as well as certain geographical information, and automating this process would allow the geographical origin of kimchi samples to be identified within 10 min.

## Conclusion

In this study, a rapid, reliable MALDI-TOF MS method, integrating multivariate analysis, was employed for the discrimination of fermented Korean and Chinese kimchi samples by geographical origin. Twenty-two peaks extracted from the mass spectra on the basis of the *q*-value (<0.2) for discrimination between Chinese and Korean kimchi samples were employed for HCA, which allowed clear differentiation between Chinese and Korean kimchi groups within 10 min. Furthermore, our MALDI-TOF MS method could be applied to discriminate the geographical origin of other fermented-salted vegetables at reduced cost in shorter times.

## Supporting information

S1 Fig Various viewpoints of the scatter plots obtained by PCA of the mass spectra of Korean and Chinese kimchi during fermentation obtained using the Mass-Up software. (PPTX)

S2 Fig Changes in the mass spectra (2,000–10,000 m/z) of Chinese and Korean kimchi samples during fermentation. Mass spectra of Korean kimchi samples after fermentation for (A) 1 week, (B) 2 weeks, (C) 3 weeks, and (D), 4 weeks; and Chinese kimchi samples after fermentation for (E) 1 week, (F) 2 weeks, (G) 3 weeks, and (H), 4 weeks. Superimposed spectra are shown for each sample corresponding to the raw data for triplicate measurements.
(PPTX)
