# Supplementary material for: A novel method to rapidly distinguish the geographical origin of traditional fermented-salted vegetables by mass fingerprinting
Source: PLoS One. 2017 Nov 17;12(11):e0188217. doi: 10.1371/journal.pone.0188217 (PMC5693415; doi:10.1371/journal.pone.0188217)
Supplement: S1 Fig — (PPTX) [file pone.0188217.s001.pptx]

## Slide 1
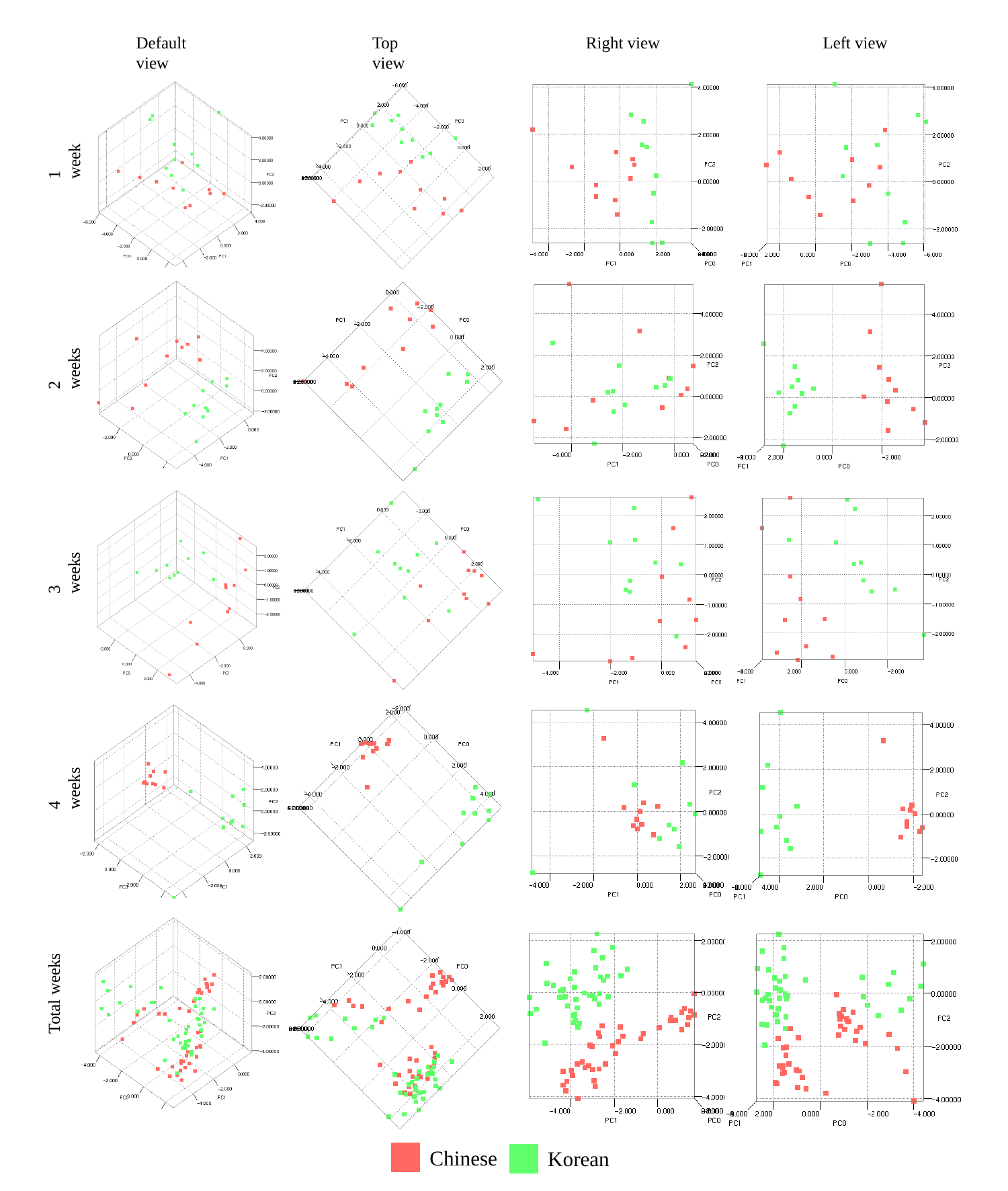

Default view
Left view
Right view
Top view
1 week
2 weeks
3 weeks
4 weeks
Total weeks
Chinese
Korean
