# Supplementary material for: A novel method to rapidly distinguish the geographical origin of traditional fermented-salted vegetables by mass fingerprinting
Source: PLoS One. 2017 Nov 17;12(11):e0188217. doi: 10.1371/journal.pone.0188217 (PMC5693415; doi:10.1371/journal.pone.0188217)
Supplement: S2 Fig — Mass spectra of Korean kimchi samples after fermentation for (A) 1 week, (B) 2 weeks, (C) 3 weeks, and (D), 4 weeks; and Chinese kimchi samples after fermentation for (E) 1 week, (F) 2 weeks, (G) 3 weeks, and (H), 4 weeks. Superimposed spectra are shown for each sample corresponding to the raw data for triplicate measurements. (PPTX) [file pone.0188217.s002.pptx]

## Slide 1
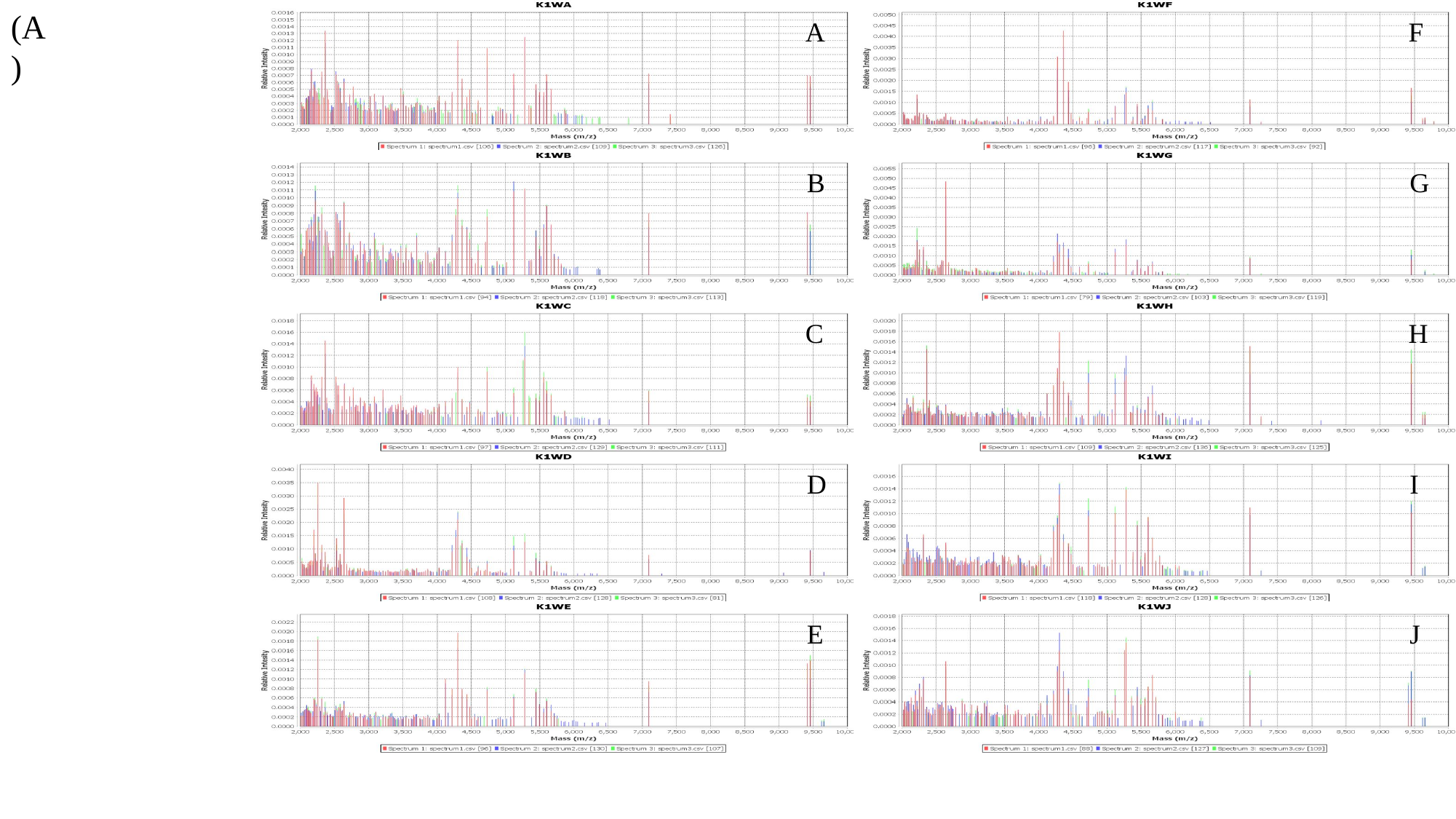

(A)
A
F
B
G
C
H
D
I
E
J

## Slide 2
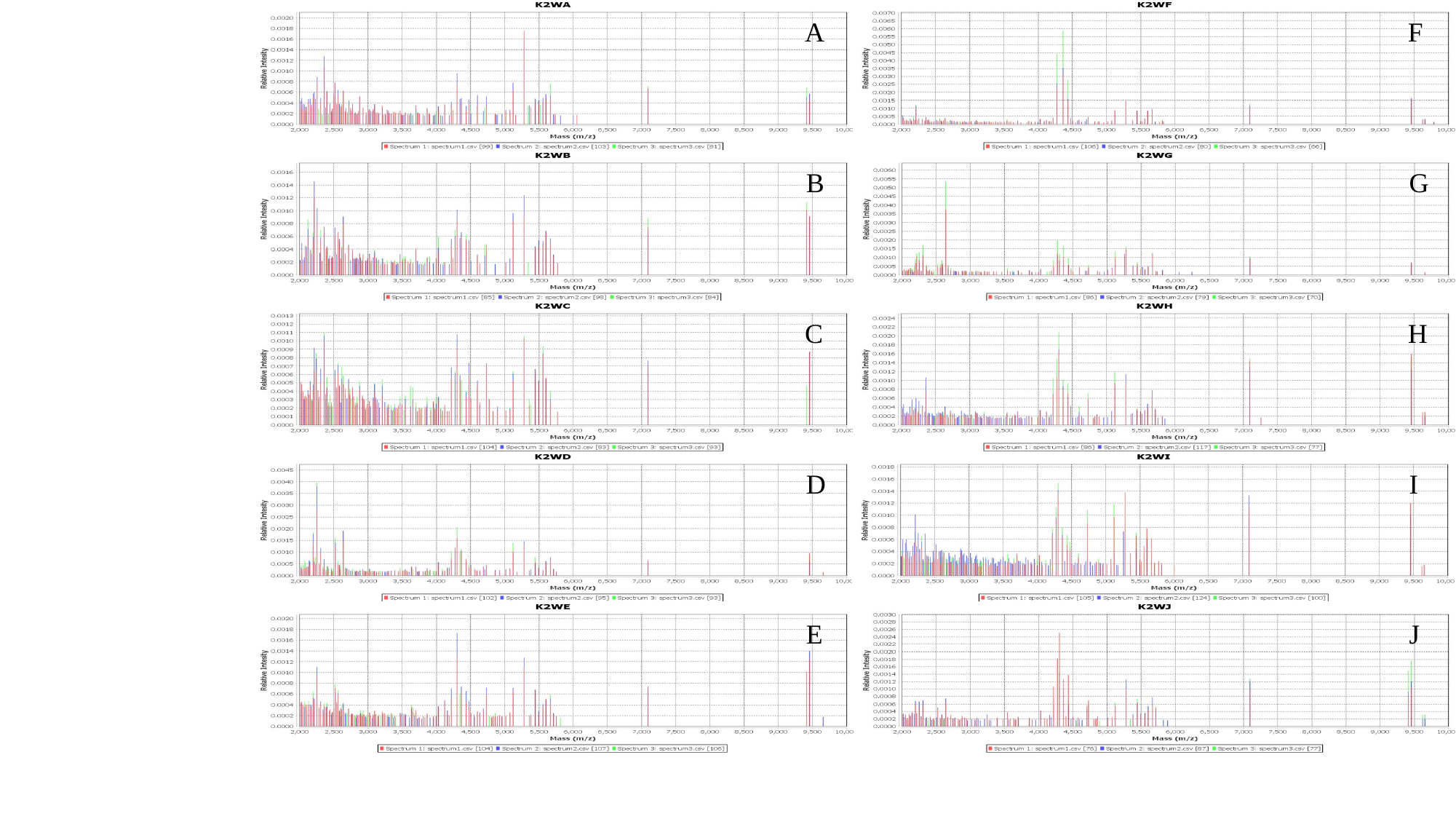

A
F
B
G
C
H
D
I
E
J

## Slide 3
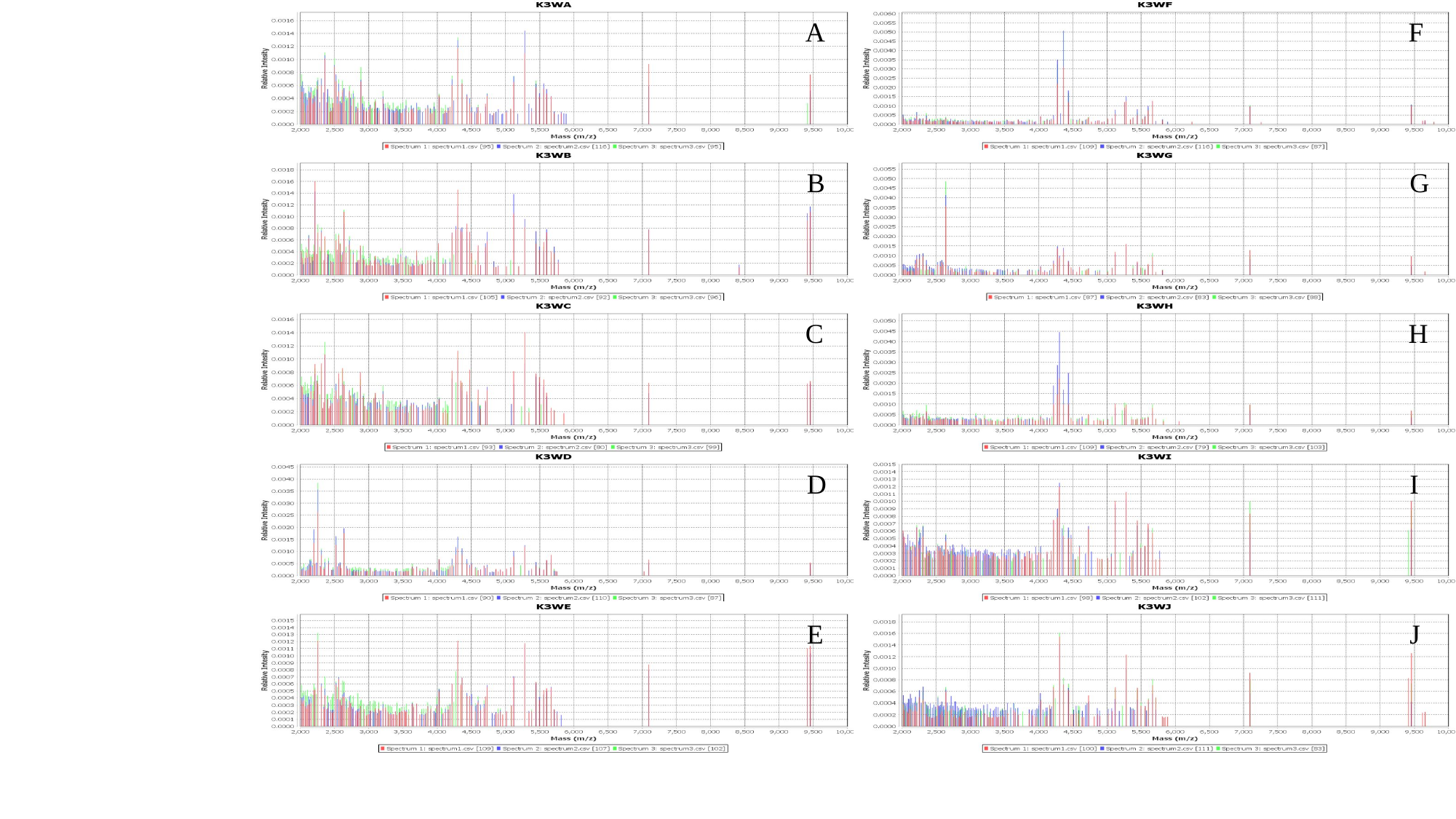

A
F
B
G
C
H
D
I
E
J

## Slide 4
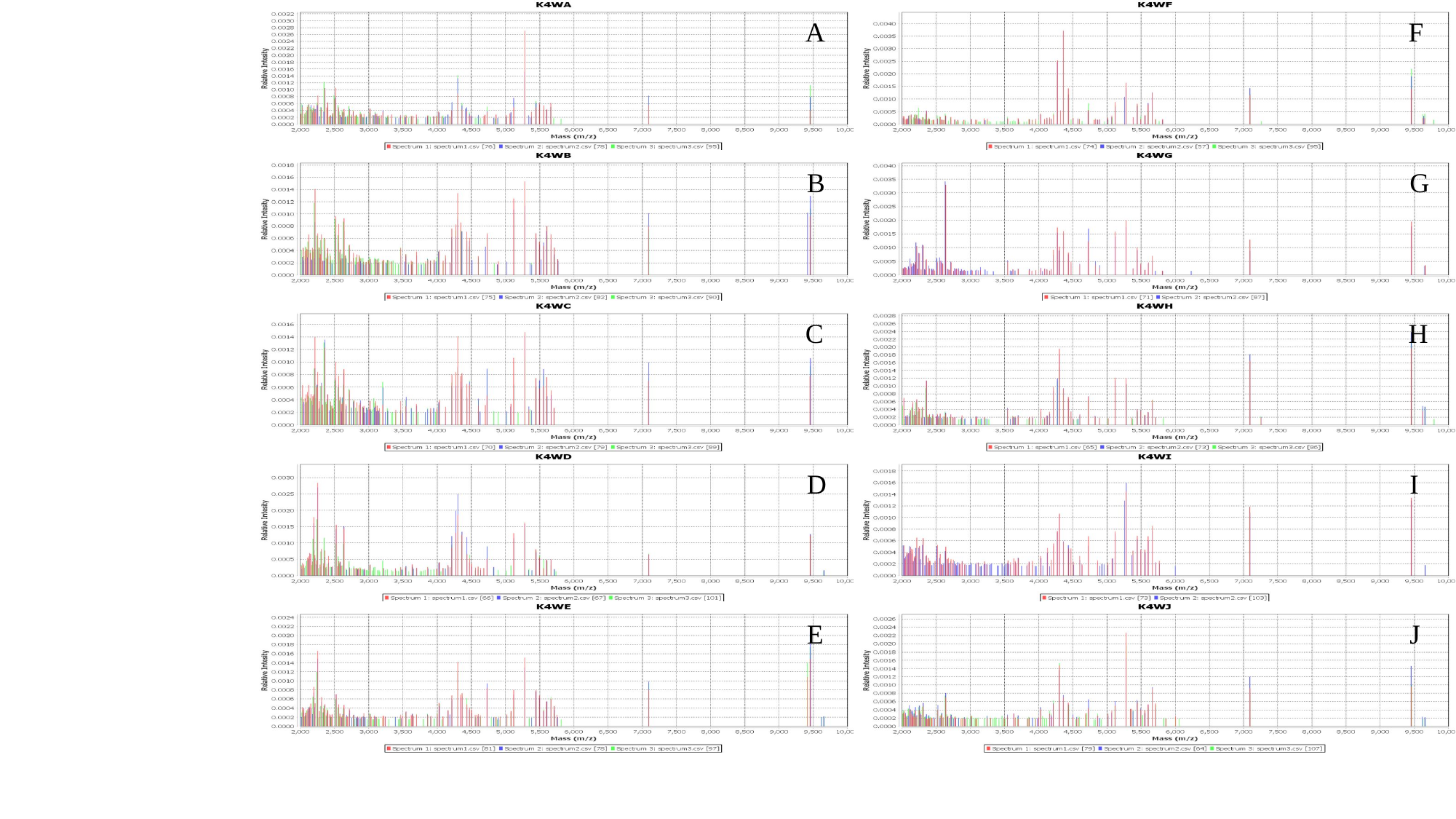

A
F
B
G
C
H
D
I
E
J

## Slide 5
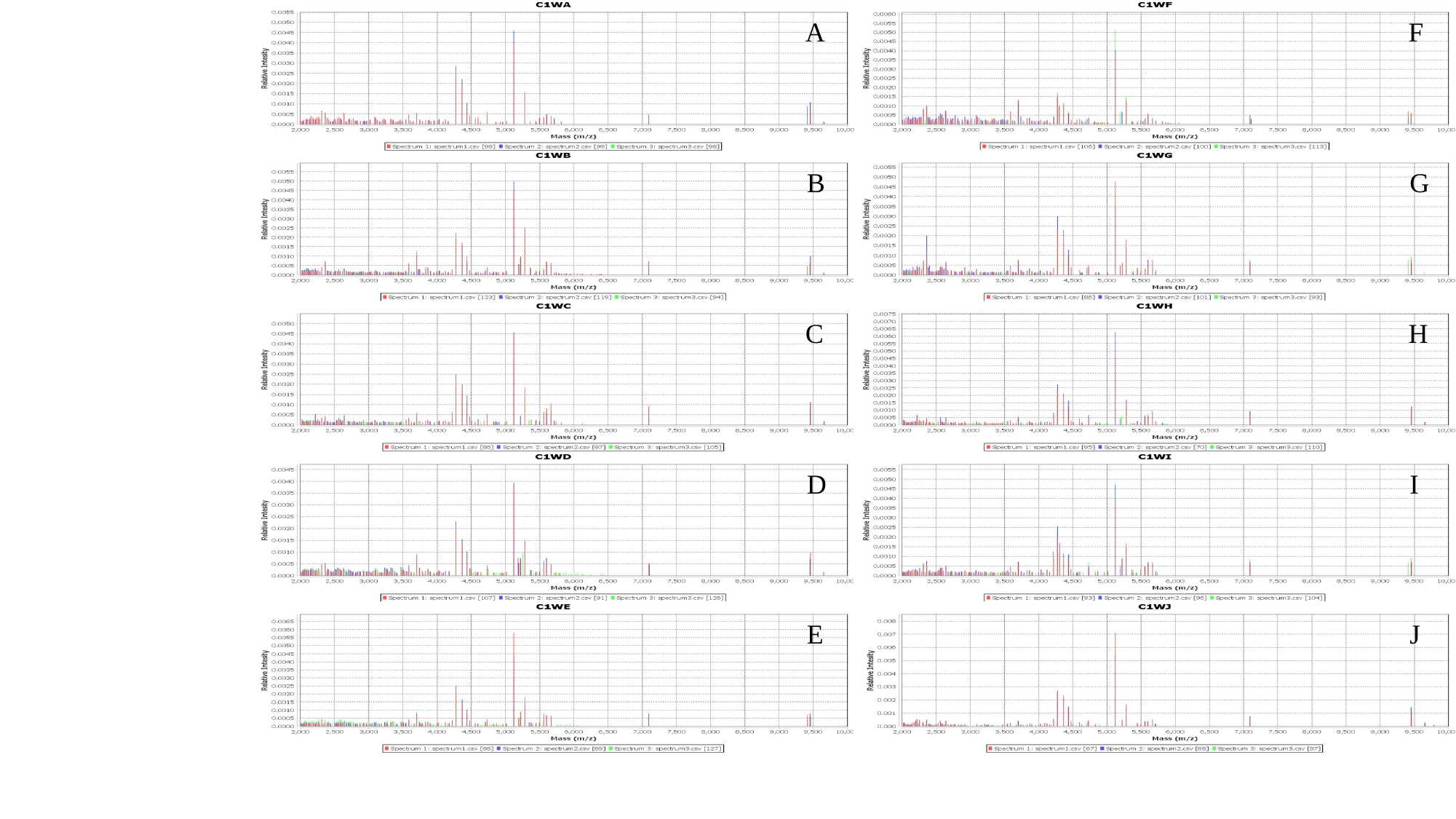

A
F
B
G
C
H
D
I
E
J

## Slide 6
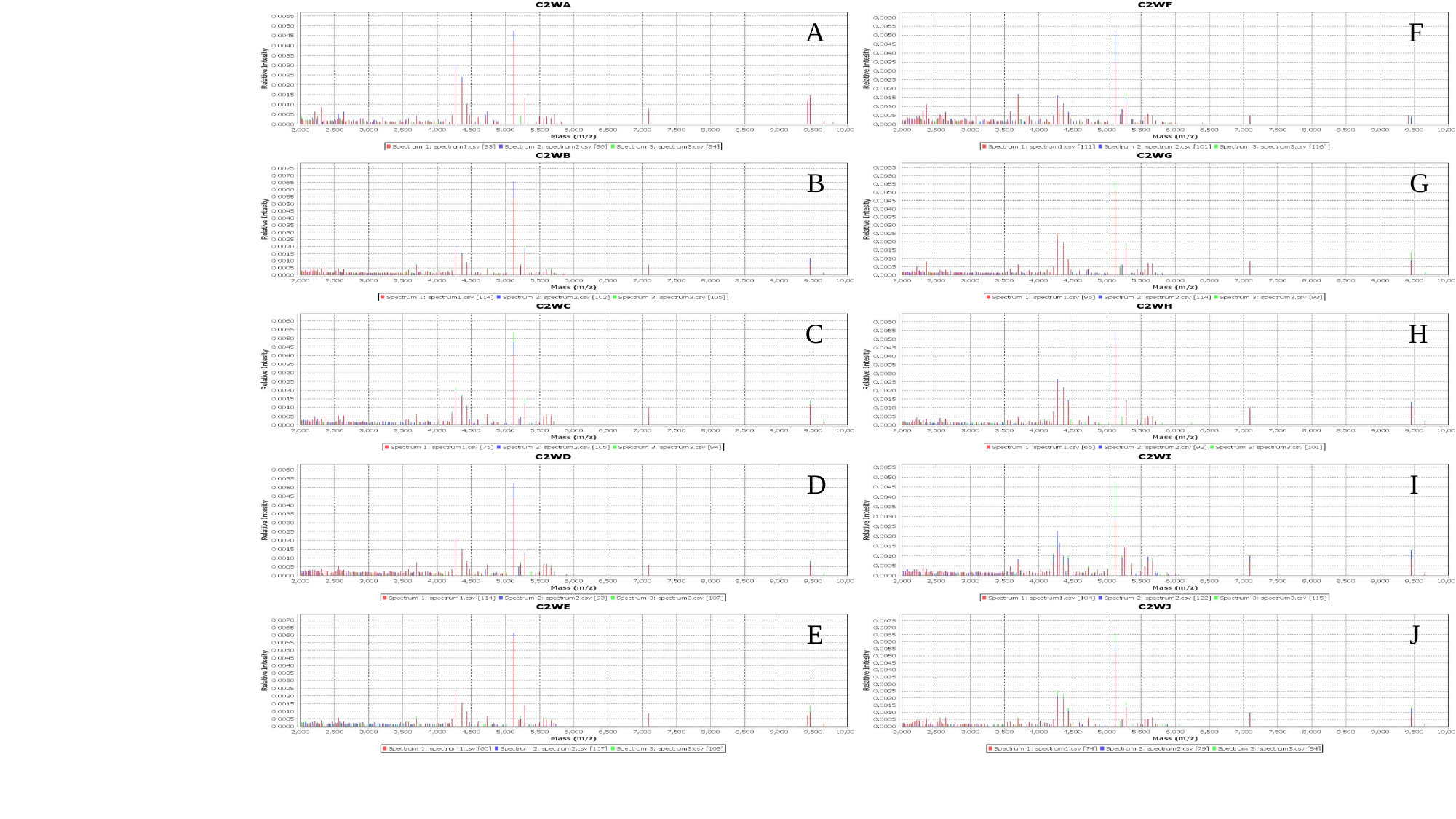

A
F
B
G
C
H
D
I
E
J

## Slide 7
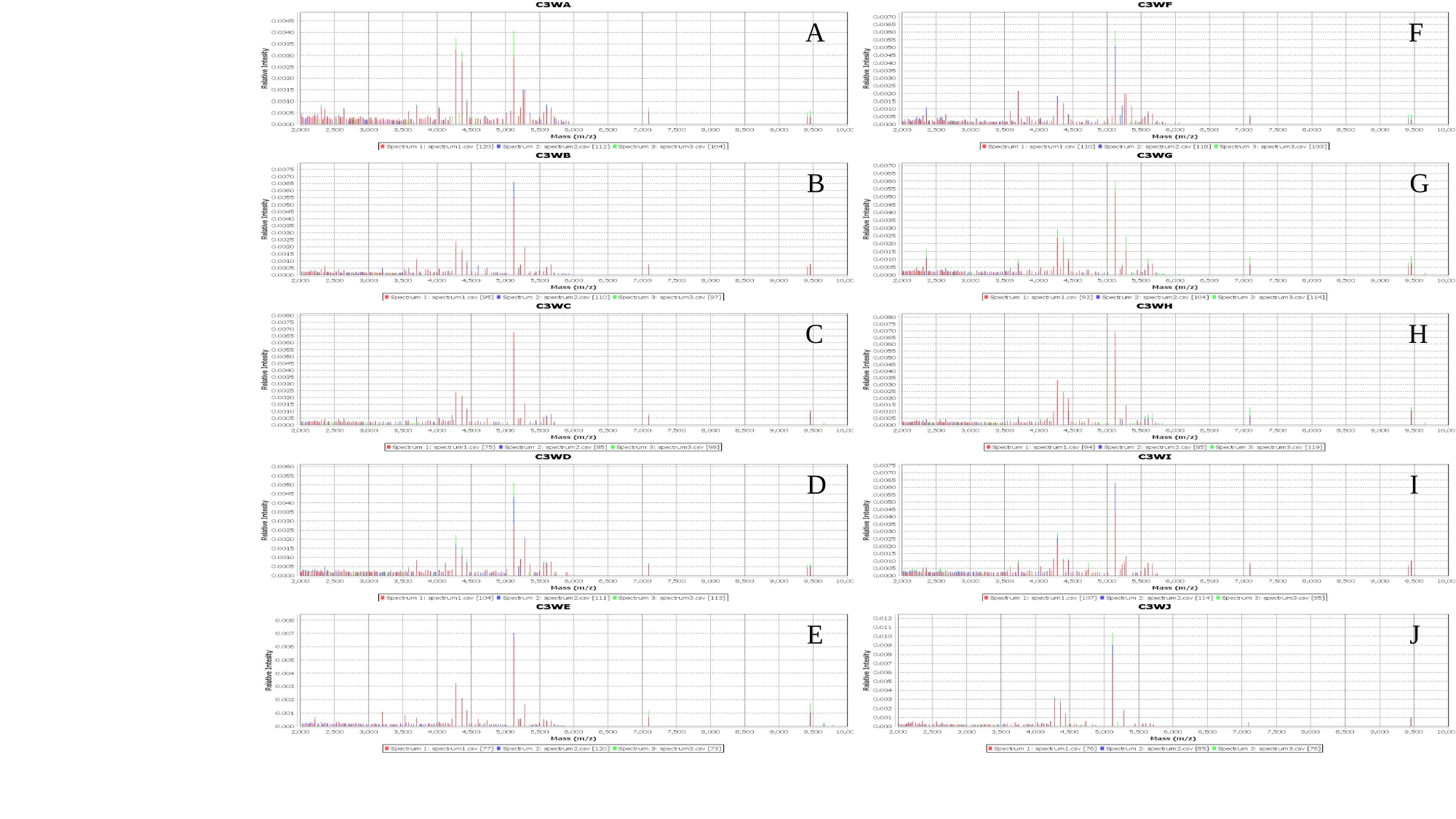

A
F
B
G
C
H
D
I
E
J

## Slide 8
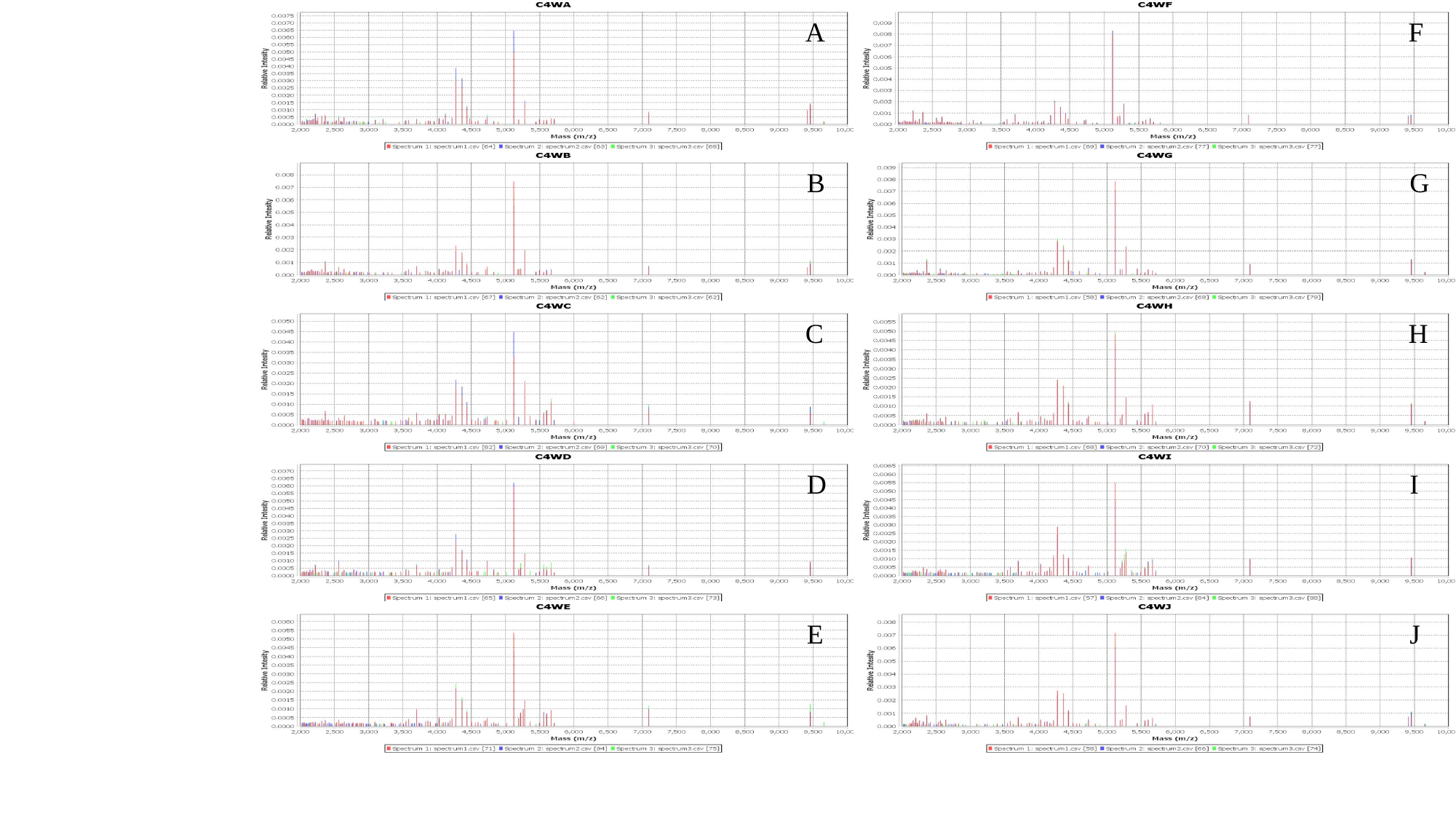

A
F
B
G
C
H
D
I
E
J
